# The PPAR-Platelet Connection: Modulators of Inflammation and Potential Cardiovascular Effects

**DOI:** 10.1155/2008/328172

**Published:** 2007-12-24

**Authors:** S. L. Spinelli, J. J. O'Brien, S. Bancos, G. M. Lehmann, D. L. Springer, N. Blumberg, C. W. Francis, M. B. Taubman, R. P. Phipps

**Affiliations:** ^1^Department of Pathology and Laboratory Medicine, University of Rochester Medical Center, 601 Elmwood Avenue, Box 608, Rochester,NY 14642,USA; ^2^Department of Environmental Medicine, University of Rochester Medical Center, 601 Elmwood Avenue, Rochester,NY 14642, USA; ^3^Cell Biology and Biochemistry, K4-12, Biological Sciences Division Battelle, Pacific Northwest Division, 902 Battelle Blvd, Richland, WA 99352, USA; ^4^M&D-Hematology/Oncology, University of Rochester Medical Center, 601 Elmwood Avenue, Rochester, NY 14642, USA; ^5^Department of Medicine, M&D-Cardiology Unit, University of Rochester Medical Center, 601 Elmwood Avenue, Box 679-ccmc, Rochester, NY 14642, USA

## Abstract

Historically, platelets were viewed as simple anucleate cells responsible for initiating thrombosis and maintaining hemostasis, but clearly they are also key mediators of inflammation and immune cell activation. An emerging body of evidence links platelet function and thrombosis to vascular inflammation. peroxisome proliferator-activated receptors (PPARs) play a major role in modulating inflammation and, interestingly, PPARs (PPARβ/δ and PPARγ) were recently identified in platelets. Additionally, PPAR agonists attenuate platelet activation; an important discovery for two reasons. First, activated platelets are formidable antagonists that initiate and prolong a cascade of events that contribute to cardiovascular disease (CVD) progression. Dampening platelet release of proinflammatory mediators, including CD40 ligand (CD40L, CD154), is essential to hinder this cascade. Second, understanding the biologic importance of platelet PPARs and the mechanism(s) by which PPARs regulate platelet activation will be imperative in designing therapeutic strategies lacking the deleterious or unwanted side effects of current treatment options.

## 1. INTRODUCTION

Cardiovascular disease (CVD) is the leading cause of morbidity and mortality world-wide. In part, this is due to social and economic changes that lead to atherosclerosis, obesity, hypertension, dyslipidemia, and type 2 diabetes mellitus (T2DM) [1–5]. Life-style factors such as exercise, healthy diet, and avoidance of smoking are crucial to prevent disease or reduce cardiovascular risk factors. While it is important to educate individuals about healthy life-style decisions, it is also imperative to develop therapeutic strategies to attenuate the chronic inflammatory pathways linked to vascular disease [4–6]. Recently, platelets have been implicated as key contributors to the chronic inflammation that leads to CVD [5].

While platelets are essential for hemostatic regulation, new studies reveal an expanded role for platelets in thrombosis, immune cell activation, and inflammatory processes creating an obvious link between thrombosis and vascular inflammation. Platelet hyperactivity is implicated in a variety of conditions including atherosclerosis, peripheral arterial disease (PAD), T2DM, and inflammatory bowel disease (IBD) [7–10]. Although activated platelets release many proinflammatory mediators such as CD40 ligand (CD40L, CD154) and thromboxane A_2_(TXA_2_), they also release membrane vesicles and platelet microparticles (PMPs), which influence the activities of other cell types both regionally and systemically. Since PMPs contain proteins important for both hemostasis and inflammation, they may amplify or sustain inflammation and thrombosis contributing to a chronic inflammatory state. Moreover, higher than normal levels of platelet-released microparticles are present in individuals with atherosclerosis, T2DM, stroke, and PAD [9, 11–13].

Proteomic studies are beginning to reveal the remarkable diversity of platelet proteins and have identified proteins not known to be expressed in or released from platelets [14–16]. While lacking a nucleus, platelets contain transcription factors, notably the peroxisome proliferator-activated receptors (PPARS). PPARs are key regulators of metabolism and inflammation, and thus are poised to play an important role in processes that govern chronic inflammatory diseases [17]. Accumulating evidence suggests that PPAR activation is beneficial in the prevention of stroke and myocardial infarction (heart attack) [17, 18]. However, other studies show that some PPAR activating drugs may increase the risk of cardiovascular events [19]. Despite the lack of definitive information on the risk and benefits of taking PPAR-targeting drugs, it is clear that PPARs remain a promising target for treating CVD and more importantly, that dampening unwanted platelet activation will reduce the risk of CVD and/or improve disease outcome.

## 2. PLATELETS ARE MODULATORS OF INFLAMMATION AND THROMBOSIS

Platelets are anuclear cells released from megakaryocytes, a hematopoietic cell that differentiates and undergoes endomitosis [20]. The platelet's composition is a product of specific packaging by the megakaryocyte and the acquisition by endocytosis of blood components. Platelets contain classical cellular organelles including mitochondria and lysosomes, a complex cytoskeleton, specific platelet granules, and an open canalicular system, a complex structure of internal membranes that serves as a conduit for the movement and release of platelet contents. Despite the lack of a nucleus, platelets contain mRNA and spliceosomal components for mRNA processing, as well as the translational machinery for protein synthesis [21–23]. The recent discovery of *de novo* synthesis by platelets of mRNAs, including Bcl-3, interleukin-1β (IL-1β), plasminogen activator inhibitor-1 (PAI-1), and tissue factor (TF), exemplifies the complexity of platelet signaling and underscores their role as formidable players in regulating coagulant and inflammatory pathways [24–29].

Platelets contain vast stores of bioactive mediators including thromboxanes, prostaglandins, chemokines, and cytokines that promote clot formation and incite inflammation. Upon activation, platelets produce high levels of proinflammatory mediators such as CD40L, intercellular adhesion molecule-1 (ICAM-1), tissue factor, and C-reactive protein (CRP). These mediators enhance inflammatory responses and recruitment of immune cells. Recently, it was shown that plasma levels of soluble CD40L (sCD40L) are high at birth and remain so throughout childhood [30]. The reason for the developmental change is not yet understood. In contrast, higher than normal adult levels of sCD40L in the adult bloodstream are linked with increased risk for ischemia, stroke, and myocardial infarcts due to thrombosis [4, 31]. Based on these studies, much interest has been generated in CD40L as a possible biomarker and major factor in the progression of CVD [32–34].

### 2.1. CD40L is a major contributor to chronic inflammation

**Figure 1:**
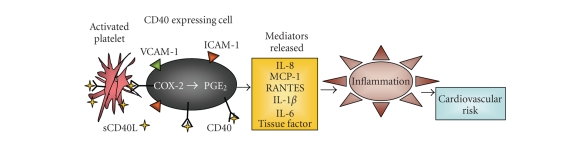
Platelets promote inflammation. CD40 expressing cells, such as endothelial cells or fibroblasts, can be activated by platelet-derived CD40L. CD40 signaling upregulates bioactive mediators in these cells; therefore, potentiating inflammation and increasing the risk for CVD.

A surprising and important finding was that CD40L, a member of the tumor necrosis factor (TNF) receptor superfamily and a key mediator of both innate and adaptive immunity [4, 5, 35, 36], is released by activated platelets [31, 33, 35]. Shortly after platelets become activated, they express CD40L on their surface which is subsequently enzymatically cleaved releasing soluble bioactive CD40L into the bloodstream. This is highly significant for the following two reasons. First, platelets contain approximately 95% of the CD40L found in human beings, and thus are a crucial link in the regulation of the CD40/CD40L pathway, as many cells express its receptor, CD40. These cells include fibroblasts, endothelial, epithelial, monocytes, neutrophils, B cells, and dendritic cells. CD40L is found in abnormally high levels in the blood of patients with chronic inflammatory diseases such as diabetes, atherosclerosis, as well as some recipients of platelet transfusions [33, 37–40]. Disruption of CD40/CD40L pathway can blunt chronic inflammation, retard atherosclerosis, and transplant rejection [33, 35, 41]. Further, recent exciting research demonstrates that CD40L is crucial for stabilizing thrombi, for normal platelet responses to sheer stress, and for platelet activation through the RGD domain of sCD40L which binds to platelet αIIbβ3, a receptor critical for platelet activation and aggregation [42, 43]. Collectively, these data strongly support the importance of CD40L as a primary agonist for platelets and is considered a prototypical mediator with roles in both hemostasis and inflammation (Figure 1 summarizes CD40 activation by platelet CD40L). Therefore, the platelet is a crucial link in the CD40/CD40L pathway and sCD40L release alone or in combination with other proinflammatory mediators may increase the risk for cardiovascular effects promoting atherosclerosis, hypertension, and dyslipidemia to list a few.

### 2.2. Platelet-released microparticles are elevated in individuals with chronic inflammatory disease

Platelet microparticles (PMPs) are defined as microvesicle particles that measure less than 1 *μ*m in diameter [44]. Platelet agonist stimulation or high shear stress leads to the highly regulated formation and release of PMPs, which are known to regulate a broad spectrum of physiological activities [45–47]. PMPs are an important delivery and cell signaling system in both inflammatory and hemostatic processes. For example, a portion of platelet IL-1*β* is associated with PMPs and signals endothelial cells, inducing their adhesiveness for neutrophils to elicit an inflammatory response [25]. PMPs signal the expression of specific adhesion molecules and stimulate the production of cytokines and mRNA in endothelial cells and in the monocytic cell line, THP-1 [48]. Notably, a known *α*-granule component and proinflammatory mediator, regulated on activation, normal T-cell expressed and secreted (RANTES) (CCL5), is delivered to sites of arterial injury and atherosclerotic endothelium via PMP to promote monocyte recruitment [49]. PMPs modulate cell-to-cell interactions by increasing adhesive contacts between monocytes and endothelial cells, an important first step in vascular inflammation [50]. It is also known that platelet-derived tissue factor (TF) is transferred from CD62P positive PMPs to monocytes although the procoagulant role of this particle delivery system has not been established [51]. Elevated numbers of PMPs are present in a variety of diseases including atherosclerosis and other CVDs, T2DM, and cancer [49, 51–54]. PPARs may have a potential role in the regulation of platelet activation and release of platelet contents as will be discussed further below.

## 3. PEROXISOME PROLIFERATOR-ACTIVATED RECEPTORS (PPARs) AND PLATELETS

PPARs are ligand-activated transcription factors and members of the nuclear hormone receptor superfamily. These receptors are known to play a role in regulating metabolic risk factors for CVD, such as the vascular inflammation and thrombosis associated with atherosclerosis [55]. There are three PPAR subtypes, PPAR*α* (NR1C1), PPAR*β*/*δ* (NUC1, NR1C2), and PPAR (NR1C3), encoded by separate genes and described in several organisms including humans. PPARs are differentially expressed in a variety of tissues and are important in the regulation of lipid and carbohydrate metabolism, energy homeostasis, cellular differentiation and apoptosis, and immune and inflammatory responses [42]. PPAR*α* is highly expressed in brown adipose tissue, liver, kidney, heart, and skeletal muscles [61]. PPAR*β*/*δ* has a broad tissue distribution with highest expression in the kidney, gut, and heart [42, 62]. PPAR*γ* is abundant in adipose tissue, colon, retina, and in cells of the immune system [58]. Important for this discussion are PPAR*β*/*δ* and PPAR*γ* as they were recently found to be expressed in human platelets, a surprising result considering platelets are anucleate [63, 64]. The impact of this discovery was exemplified upon finding that exposure to PPAR agonists attenuates platelet activation and associated inflammation [63, 64].

Activation of PPARs in nucleated cells occurs by optimal DNA binding to a PPAR DNA response element following ligand binding and conformational changes that facilitate heterodimerization with a second ligand-activated nuclear receptor, retinoic X receptor (RXR, 9-cis retinoic acid receptor) [65, 66]. This heterodimer binds to a cis acting DNA element in the promoters of target genes called the peroxisome proliferator response element (PPRE) to induce or repress gene transcription in a cell- and tissue-specific manner, depending on the receptor and a combination of factors, including ligand and accessory molecule binding. The physiological functions of PPAR*α* and PPAR*γ* have been relatively well characterized, whereas the function of PPAR*β*/*δ* is poorly understood. A summary of the PPAR subtypes and their potential roles in platelets is discussed below.

### 3.1. PPAR*α*

PPAR*α* activation affects transcriptional expression of approximately 80–100 genes, the products of which regulate fatty acid oxidation, lipid metabolism, and inflammation [67]. PPAR*α* is expressed in cells of the vasculature and immune system, but has not yet been firmly identified in platelets [68]. The antiinflammatory properties of PPAR*α* are of paramount interest, but there are also reports of proinflammatory effects [69, 70]. For example, it was demonstrated that chronic activation of PPAR*α* is detrimental to cardiac recovery during reperfusion following ischemia [71]. In contrast, it is known that PPAR*α* plays an antiinflammatory role in lung fibrosis although the mechanism is not well understood [72, 73]. It is clear that the intricacies of PPAR*α* function must be discerned to design effective and safe drug strategies. Current PPAR*α* agonists include the fibrates, which are therapeutic agents that increase transcription of high density lipoproteins (HDL) such as ApoAI and ApoAII and are effective at lowering triglyceride levels [74, 75]. PPAR*α* agonists have also been reported to decrease weight gain, as obesity is a contributing factor in atherosclerosis [75].

### 3.2. PPAR*β*/*δ*

PPAR*β*/*δ* is suggested to play a role in basic cellular functions such as cellular proliferation and differentiation, and fatty acid catabolism in skeletal muscle where it is most abundant [76, 77]. This receptor has also been implicated in the regulation of inflammation, and shown to slow plaque formation and attenuate the progression of atherosclerosis [78]. Although little is known about the function of PPAR*β*/*δ*, especially in platelets, prostacyclin (PGI_2_), an important antithrombotic and endogenous platelet hormone, is reported to be a ligand for PPAR*β*/*δ* [79, 80]. Several studies have revealed that PGI_2_ synergizes with nitric oxide (NO) to inhibit platelet aggregation in response to a variety of platelet agonists including thrombin, collagen, ADP, and lysophosphatidic acid (LPA) [64, 81–86]. It was previously shown that the synergistic effects of NO and prostacyclin on inhibition of platelet response were due to the simultaneous increase of cyclic nucleotides cGMP and cAMP [81, 87, 88]. The recent discovery that PPAR*β*/*δ* ligands and NO inhibit platelet aggregation via PPAR*β*/*δ* suggests an alternative signaling mechanism is operative in platelets [64]. This is consistent with a previous study where Ali et al. demonstrated that prostacyclin mimetics exhibited antiproliferative effects that were mediated by PPAR*β*/*δ* and not via the prostacyclin receptor in lung fibroblasts [89]. This identified PPAR*β*/*δ* as a potential therapeutic target for the treatment of pulmonary hypertension and supports the view that platelet PPAR*β*/*δ* may play an important role in thrombosis [64].

### 3.3. PPAR*γ*

PPAR*γ* is important in adipocyte differentiation, lipid storage, and glucose homeostasis, and has emerged as a key target for new anti-inflammatory therapies [6, 90, 91]. There are 3 isoforms of PPAR*γ* (PPAR*γ*1, PPAR*γ*2, and PPAR*γ*3). All are encoded by the same gene, but are the result of differential promoter use and alternative RNA splicing [92]. PPAR*γ*2 differs from PPAR*γ*1 by an additional 30 amino acids at the N-terminus. PPAR*γ*1 is present in adipose tissue, human spleen, liver, intestine, kidney, and platelets, while PPAR*γ*2 is abundantly expressed only in adipose tissue and liver [93]. PPAR*γ*3 mRNA has been detected in mouse macrophage cells, however its function remains unknown [94].

PPAR*γ* is expressed in many cell types including fibroblasts, endothelial cells, dendritic cells, macrophages, T cells, B cells, and most recently we identified PPAR*γ* in human platelets [59, 63, 91, 95–98]. Our laboratory recently discovered that human platelets express PPAR*γ* and that PPAR*γ* ligands attenuate platelet release of the proinflammatory and procoagulant mediators, sCD40L and TXA_2_, a cyclooxygenase (COX) product that enhances platelet activation [63]. Platelets can respond to at least two natural PPAR*γ* ligands: lysophosphatidic acid (LPA) which they produce, and 15d-PGJ_2_ which has potent antiinflammatory properties and is a metabolite of PGD_2_ [91, 99, 100]. Additionally, there are several synthetic ligands in development and clinical use that are specific and potent agonists for PPAR*γ* including the antidiabetic thiazolidinedione drugs (TZDs) (e.g., rosiglitazone (Avandia) and pioglitazone (Actos) both in clinical use) [91, 99]. These will be discussed in greater detail in Section 5.

Interestingly, human platelets also contain the PPAR*γ* binding partner RXR, and PPAR*γ* is able to bind DNA suggesting that it can form an active PPAR*γ*/RXR heterodimer, and thus may be capable of biologic activity within the platelet. It is therefore possible that PPAR*γ* agonists interact directly with platelets to alter platelet activation and hemostatic function. While PPAR*γ* was first thought to be located only in the nucleus to regulate transcription, we and others have demonstrated that PPAR*γ* can be found in the cytoplasm of eukaryotic cells [91, 101]. There is increasing evidence suggesting that PPAR*γ* binds proteins in the cytoplasm of cells separate from its transcriptional role. For example, it was recently reported that PPAR*γ* ligands, via a PPAR*γ*-dependent mechanism, block PKC*α* translocation to the membrane attenuating inflammatory responses in monocytes/macrophages [101]. Additionally, cytoplasmic PPAR*γ* can repress the transcriptional activity of the proinflammatory mediator, nuclear factor–κB (NF-κB), preventing its translocation to the nucleus [92, 102]. NF-κB is involved in regulating many aspects of cellular activity, including the immune response and has a well established role in the pathological progression of chronic inflammatory diseases [103]. Interestingly, it has also been shown in platelets that the PPAR*γ* binding partner, RXR, signals through the Gq-protein receptor in a ligand-dependent manner to inhibit platelet activation [104].

Intriguingly, our group has discovered that PPAR*γ* is released in a PMP-associated form and some PPAR*γ* is expelled from activated platelets as a functional PPAR*γ*/RXR heterodimer [105]. Moreover, the released PPAR*γ* is taken up by a promonocytic cell line (THP-1) [105]. Thus, it is possible that other cells also take up platelet-released PPAR*γ*, quickly elevating PPAR*γ* levels in recipient cells. This potential transcellular mechanism for PPAR*γ* would then influence the recipient cell's susceptibility to PPAR*γ* ligands and may represent a novel antiinflammatory mechanism. For example, PPAR*γ* and its ligands are known to reduce VCAM-1 and ICAM-1 expression, and increase nitric oxide synthase expression on endothelial cells which is important for inhibiting platelet activation [106, 107]. These expanded antiinflammatory roles for PPAR*γ* provide new avenues to pursue novel drug strategies.

## 4. PLATELETS AND CARDIOVASCULAR DISEASE

Cardiovascular disease comprises a broad spectrum of illnesses, such as hypertension, dyslipidemia, and myocardial infarction and stroke that affect the heart and the blood vessels. These conditions have similar causes (obesity, smoking, diabetes, sedentary lifestyle, and age) and platelets play a complex role in CVD, triggering early events that lead to endothelial dysfunction, to progression of vascular damage, to plaque production, and to formation of thrombi that can result in myocardial infarcts and stroke.

### 4.1. Metabolic syndrome

Platelets and their PPARs play putative roles in several manifestations of the dyslipidemia-associated “metabolic syndrome” or “syndrome X,” which includes hyperglycemia, insulin resistance, obesity, hypertension, and atherosclerosis [77, 108–113]. Dyslipidemia, an increasingly common consequence of a high-fat diet, is characterized by increased serum triglycerides, low levels of antiatherogenic high density lipoprotein cholesterol (HDL) and prevalence of pro-atherogenic low density lipoprotein particles (LDL). Considering the imbalance between pro- and antiatherogenic factors, it is not surprising that dyslipidemia is associated with a high risk of atherosclerosis in afflicted patients [77]. HDL protects against atherosclerosis by driving the reverse transport of cholesterol from peripheral cells to the liver for excretion [77, 113]. The contribution of LDL particles to the development of atherosclerosis is closely connected to platelet function and may be modulated by PPARs, as described below.

### 4.2. Atherosclerosis

Atherosclerosis is a chronic inflammatory disease characterized by plaque development within the arterial intima [5, 114]. These atherosclerotic plaques may erode or rupture over time, triggering thrombogenesis, and possible myocardial infarction or stroke [5, 115]. Platelets are famous for their role in clot formation during the final stages of atherosclerosis, but it has become clear from studies in both humans and animal models that the early stages of plaque formation are also platelet-mediated [5, 115–120]. Atherosclerosis is initiated when inflammatory processes activate vascular endothelial cells, resulting in platelet adhesion to the arterial wall [115, 121–123]. When platelets adhere to the endothelial surface, they are activated, causing them to release mediators that attract and activate other cell types, including neutrophils, monocytes, and bone-marrow-derived progenitor cells [5, 115]. Monocytes cross the endothelial monolayer and enter the arterial intima by extravasation [115]. There they differentiate first into macrophages, and then, into cholesterol-laden foam cells, a critical step in atherosclerotic plaque formation [77, 115, 118]. Platelets regulate the differentiation of bone-marrow-derived progenitor cells and macrophages into foam cells [5, 115, 118, 119, 124]. Studies using fluorochrome-modified LDL have shown that platelets take up LDL and store it in dense granules [115, 118]. These platelets can then be internalized by macrophages, a critical step in foam cell differentiation and plaque formation [115, 118, 125, 126].

One platelet-derived mediator with a clear link to atherogenesis is platelet factor 4 (PF4) which both inhibits LDL degradation by the LDL receptor and promotes monocyte-to-foam cell differentiation [115, 127]. Activated platelets also release CD40L and interleukin-1*β* which further activate the vascular endothelium, causing it to produce chemoattractants and adhesion molecules that act to recruit neutrophils and monocytes into the arterial intima [5, 115, 118, 128, 129]. Matrix metalloproteinases (MMPs) are also expressed by activated platelets, monocytes, and endothelial cells in response to CD40L; these are important in foam cell generation and the physical remodeling of the normal arterial wall to an atherosclerotic plaque [115, 118, 130–136]. Smooth muscle cell proliferation, promoted by platelet release of transforming growth factor-*β*, platelet-derived growth factor, and serotonin, is also critical to this process [115].

PPARs appear to play a major role in the regulation of atherogenesis by countering the inflammation-provoking action of platelet adhesion and activation [5]. In vitro incubation of platelets with PPAR*γ* agonists inhibits their ability to express CD40L and to aggregate in response to thrombin [63, 137]. Pioglitazone, a PPAR*γ*-specific ligand, decreases platelet aggregation and delays arterial thrombus formation in male LDL receptor-deficient mice [5, 138]. Other PPAR*γ* ligands, including rosiglitazone and c9, t11-conjugated linoleic acid, inhibit atherosclerotic progression in this model and in the apoE^−/−^ mouse [139, 140], possibly through their ability to inhibit platelet deposition, monocyte recruitment, macrophage differentiation, LDL uptake, foam cell formation, MMP expression, and vascular smooth muscle cell migration within atherosclerotic plaques [115, 118, 137, 138, 141, 142]. Studies in human patients with atherosclerosis have shown that certain TZD type PPAR*γ* agonists reduce both platelet and endothelial cell activation, inhibit plaque progression, improve flow-mediated vasodilation, and remarkably promote regression of existing atherosclerotic plaques [5, 115, 143]. Since phagocytosis of platelets (and their internalized LDL) by macrophages is critical to foam cell formation and atherosclerotic progression, platelet-derived PPAR*γ* may be of paramount importance to the antiatherosclerotic actions of these drugs [115, 118, 125, 126]. Packaging of PPAR*γ* into platelets and/or its release in PMPs may be a convenient mechanism by which this transcription factor is delivered to endothelial lesions where it may act to attenuate pathological remodeling of the arterial wall. The potential benefits of PPAR signaling are not limited to atherosclerosis, but may extend to “metabolic syndrome” as a whole. Rosiglitazone therapy reduces the systemic inflammation characteristic of “metabolic syndrome,” as evidenced by decreases in serum levels of IL-6 and TNF*α* [5, 144]. PPAR*β*/*δ* ligands have been shown to ameliorate dyslipidemia in both mice and insulin-resistant obese rhesus monkeys [113, 145, 146]. Current data suggest that PPARs will prove to be premium targets for the development of drugs to combat both dyslipidemia and atherosclerosis.

### 4.3. Thrombosis

As was discussed above, endothelial dysfunction in blood vessels is one of the earliest events that contribute to disease development triggering a chain reaction, which results in formation of atherosclerotic plaques and rupture in the blood vessel walls. A major function of platelets is to “plug” these holes by changing their shape, adhering to subendothelial surfaces, secreting the contents of intracellular organelles, and aggregating to form a thrombus in response to stimuli generated in endothelia of damaged blood vessels [147]. Several mediators are involved in platelet aggregation, such as thrombin, collagen, epinephrine (exogenous to the platelet); agents such as ADP (secreted from platelet storage granules); and thromboxane A_2_ (synthesized by the platelets during activation) [148]. As was mentioned above, the PPAR*γ* agonists rosiglitazone and pioglitazone dampened platelet release of key proinflamatory and proatherogenic mediators such as CD40L and TXA_2_ [63]. The PPAR*γ* agonist troglitazone has also been shown to decrease platelet aggregation in response to ADP, collagen, and arachidonic acid [149]. The mechanism whereby the vascular endothelium defends against thrombus formation involves the generation of the potent vasodilator nitric oxide (NO). NO interferes with platelet aggregation and is generated from L-arginine by the enzyme nitric oxide synthase (NOS) which is constitutively expressed in endothelium [150]. In experiments where rats received pioglitazone, it was found that aortic cNOS and thrombomodulin expression was upregulated and thrombus formation was delayed [151]. Pioglitazone had similar effects in the human monocyte/macrophage cell line (THP-1) where dose-dependently upregulated thrombomodulin expression was seen [152]. Other PPAR*γ* ligands, such as rosiglitazone, also upregulate cNOS gene expression [153, 154].

### 4.4. Myocardial infarction and stroke

Myocardial infarction occurs when the blood supply to the heart is interrupted causing damage and possible death of the heart tissue. One of the major causes of myocardial infarction is rupture of the atherosclerotic plaque and formation of a platelet-rich thrombus. PPAR*γ* is present in heart tissue, but there is limited data about its function there. The PPAR*γ* activator rosiglitazone does inhibit TNF-*α* gene expression in cultured myocytes [155]. Additionally, Rosiglitazone treatment of male Lewis rats following myocardial ischemia and reperfusion injury showed a dramatic protection against myocardial infarction, and also improved cardiac function [156]. Ischemia/reperfusion injury is characterized by an inflammatory response. Activated neutrophils release a variety of cytotoxic substances, such as oxygen-derived free radicals and proteases and activated monocytes/macrophages synthesize inflammatory cytokines [157]. Activated platelets can upregulate these responses in neutrophils and monocytes/macrophages. Together, these mediators directly participate in the amplification of an inflammatory response and, therefore, in vascular endothelial dysfunction that can lead to myocardial injury. PPAR*γ* is present in monocytes/macrophages, neutrophils, and platelets, which suggests a role for PPAR*γ* in negatively regulating expression of proinflammatory genes and thus, myocardial infarction [158].

Thrombus can also form in the cerebral arteries blocking the normal blood flow and causing a cerebrovascular accident (stroke). Stroke is a complex process in which several pathways are involved and successful prevention of a stroke will require drugs with pleiotropic effects. Resveratrol, found in the seeds and skin of grapes, was found to have neuroprotective effects [159] and shown to be a dual PPAR*α*/*γ* activator [18]. Experiments in a rat model have shown that pretreatment with fenofibrate and/or Wy-14643, which are PPAR*α* activators, and resveratrol reduced brain infarct size after permanent focal cerebral ischemia [18]. PPAR*β*/*δ* is found in numerous brain areas whereas PPAR*α* and PPAR*γ* have a more localized expression. Inflammation and oxidative stress induce apoptotic and necrotic neuronal death and NF-κB is one of the culprits [160]. It is thought that PPARs have a neuroprotective function due to their interaction with NF-κB. For example, PPAR*γ* binds to NF-κB complexes and facilitates its translocation out of the nucleus [102]. Due to their wide distribution in the neurovascular-glial compartments and their complex function, PPAR agonists offer hope in the prevention of stroke [161]. It will be of major importance to dampen platelet activity in the case of both myocardial infarction and stroke as ultimately, hyperactive platelets will be the major culprits in the occlusion or rupture of an artery.

### 4.5. Diabetes mellitus

Type 2 diabetes mellitus (T2DM), primarily characterized by hyperglycemia and insulin resistance, is often part of a “metabolic syndrome” which comprises hypertension, dyslipidemia, decreased fibrinolysis, and increased procoagulant factors (discussed above) [162]. Thrombocytopathia (any qualitative modification of platelets) in diabetes includes: increased platelet aggregation and adhesiveness, increased platelet number, and enhanced expression of activation-dependent adhesion molecules [10]. Platelet hyperaggregability and adhesiveness in diabetes has several causes. Prostacyclin and the endothelium-derived relaxing factor nitric oxide (NO) are released by intact vascular endothelium and antagonize the effects of proaggregants so that thrombi do not form in blood vessels [163]. Platelets from diabetic patients produce less prostacyclin and NO and, in addition, they are less sensitive to PGI_2_ and nitric oxides inhibitory effects [164–166]. Insulin can target platelets directly through the platelet insulin receptor, which binds insulin and undergoes autophosphorylation [167]. Insulin reduces platelet responses to the agonists ADP, collagen, thrombin, arachidonate, and platelet-activating factor [168]. However, in T2DM platelets express fewer insulin receptors and a decreased affinity for insulin [169]. Insulin has a direct effect on platelets and is important for maintaining platelet PGI_2_ sensitivity by increasing the PGI_2_ binding sites and as a consequence, augments cAMP response to PGI_2_ [170]. Numerous studies support the fact that there is an association between diabetes and oxidative stress [171]. A higher production of reactive oxygen species is thought to play an important role in diabetes complications and has been attributed to protein glycation and/or autoxidation caused by a hyperglycemic environment, and lipid peroxidation of cellular structures [172].

Oxidative defense is provided by vitamins, such as vitamin E, and by a number of enzymes, such as glutathione peroxidases. Platelets contain two glutathione peroxidases: cytosolic glutathione peroxidase (cGPx) and phospholipid hydroperoxide glutathione peroxidase (PHGPx). CGPx is involved in oxidative stress protection and in formation of eicosanoids [173, 174]. Vitamin E is decreased in plasma of type 1 and type 2 diabetic patients [175]. In type 2 diabetics, platelet cGPx activities were found to be lower and can lead to a relative accumulation of 12-hydroperoxy-eicosatetraenoic acid (12-HpETE), the main hydroperoxide formed from arachidonic acid [175]. Thus, increase in 12-HpETE could activate signal transduction pathways leading to arachidonic acid release, and amplification of platelet activation [176]. Platelet PHGPx activity was also measured for the first time in diabetic patients and was decreased in type 2 diabetics [175]. Thus, in diabetes there is an increase in free radical production and a decrease in mechanisms responsible for antioxidant defense which give rise to an environment that favors generation of radical species. Type 1 and 2 diabetic patients exhibit increased expression of activation-dependent adhesion molecules, such as activated *α*IIb*β*_3_, lysosomal Gp53, thrombospondin, and P-selectin (CD62P) [177]. The increased expression of *α*IIb*β*_3_ is consistent with the enhanced fibrinogen binding and aggregability seen in platelets from diabetic subjects [178]. Arachidonic acid metabolism, which leads to TXA_2_ production, is increased in diabetes and may cause platelet sensitivity [179, 180]. Because diabetes is accompanied by CVD development, drugs that can reduce hyperglycemia and inhibit the progression of cardiovascular complications are desirable. PPAR*α*/*γ*/*β* pan agonists may offer new options for treatment of diabetic complications. The blood of both type 1 and 2 diabetics shows elevated levels of CD40L [39]. PPAR*γ* ligands can reduce platelet activation and thrombosis by reducing CD40L from platelets. Treatment of diabetic patients with TZD-type drugs decreased circulating CD40L blood levels [181].

### 4.6. Obesity

Obesity represents a major health threat and, in recent years, it has become clear that obesity and inflammation are linked [109 –111, 182]. Obese individuals show persistent platelet activation and subsequent increased plasma levels of several proinflamatory cytokines [183]. TNF*α*, adiponectin, leptin, and monocyte chemoattractant protein-1, all can originate from fat, have immunomodulating functions and show an altered profile during obesity [184]. Furthermore, PPAR*β*/*δ* has been linked to the development of obesity. Its activation decreases adipose mass in mouse and increases fatty acid oxidation in the heart, improving muscle contraction [76]. Thus dampening platelet activation may be a means of reducing an inflammatory cascade that leads to further vascular damage and CVD.

## 5. PPAR AGONISTS AS PLATELET THERAPEUTICS

Platelets are an important pharmacological target because the thrombi developed during CVD that lead to morbidity and mortality are platelet-rich in content. Nonsteroidal antiinflammatory drugs, including aspirin, are among the most widely used drugs around the world [185]. Aspirin's primary action is to inhibit arachidonate-cyclooxygenase activity in platelets and ultimately, TXA_2_ release thereby, attenuating thrombus formation. Recent reports show that a subset of patients is aspirin-resistant and that aspirin may not be as effective in women. This, coupled with the fact that the cyclooxygenase pathway plays only a minor role in the action of many platelet agonists, has lead to the development of new antiplatelet therapies that complement aspirin's therapeutic effects [186–189].

There are two groups of antiplatelet agents used in conjunction with aspirin: the thienopyridines (ticlopidine and clopidogrel) and the glycoprotein (GP) IIb/IIIa (*α*IIb*β*_3_) receptor antagonists (abciximab and eptifibatide). The thienopyridines are adenosine $5^{\prime }$-diphosphate (ADP) receptor antagonists which block ADP from binding, thereby, inhibiting platelet activation, aggregation, and degranulation. While for the most part, thienopyridines are efficacious for reducing ischemic events, it is unclear as to whether or not clopidogrel and aspirin together are more effective than aspirin alone [190, 191]. In rare cases, thienopyridines may cause neutropenia or thrombotic thrombocytopenia purpura [192, 193].

*α*IIb*β*_3_ is the most important platelet membrane receptor for aggregation because it is found in high concentrations on the cell surface and binds both fibrinogen and von Willebrand factor. Blocking this receptor reduces thrombotic risks associated with acute coronary syndromes and diabetes. Unfortunately, *α*IIb*β*_3_ receptor antagonists have to be administered intravenously because oral therapy causes excessive bleeding [194]. Moreover, a meta analysis of four *α*IIb*β*_3_ receptor antagonist trials showed an overall increase in mortality with drug use [195].

Clearly, there is a need to develop new therapeutics that are easily administered and can dampen platelet function with fewer adverse side effects. Adding complexity to function, platelets activate and release many proinflammatory mediators and interact with not only each other, but also with many other cell-types as described in previous sections. Targeting this action of platelets could be effective in not only reducing platelet aggregation and thrombus formation, but also in attenuating chronic inflammation and, therefore, slowing disease progression.

**Figure 2:**
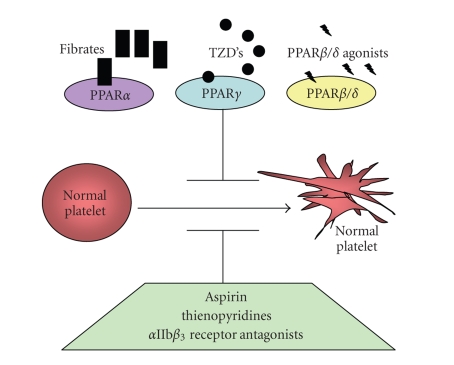
Possible role of PPAR agonists in dampening inflammation and reducing cardiovascular events. PPAR agonists may reduce the risk for thrombosis. Besides playing a role in adipogenesis, lipid metabolism, and insulin sensitivity, PPARs may dampen inflammation by attenuating platelet activation.

PPAR agonists are a class of potential antiplatelet drugs that are easily administered and have the ability to impact this new physiology of platelet function. Even though PPAR agonists are primarily prescribed for the treatment of metabolic disorders, some possess the secondary benefit of inhibiting cardiovascular complications associated with hyperlipidemia and hyperglycemia. PPAR*α* agonists, fibrates, are prescribed for hyperlipidemia. They potently diminish blood cholesterol and triglyceride levels while raising plasma HDL levels (platelet agonists that dampen platelet activation are summarized in Figure 2).

The effect of PPAR*α* agonists on cardiovascular risk during clinical studies show mixed results. The Veterans Affair High-Density Lipoprotein Cholesterol Intervention Trial study (VA-HIT) demonstrated that the fibrate, gemfibrozil, significantly reduced nonfatal myocardial infarction and death in men with coronary cardiopathy [196]. Disappointingly, results from the recent Fenofibrate Intervention and Event Lowering in Diabetes (FIELD) trial showed no reduction in risk for the primary end-point (coronary heart disease death and nonfatatal myocardial infarction) in coronary events with fenofibrate therapy [197]. There are many explanations for these results, including the use of a low cardiovascular risk diabetic population, but it is clear that more investigation is needed to understand the clinical relevance of fibrates for treating CVD. Since platelets may lack PPAR*α*, these drugs may not have a direct effect on platelet function, but may be useful in conjunction with other PPAR agonists to target multiple pathways involved in cardiovascular pathophysiology (see below).

Perhaps more promising is the use of PPAR*γ* TZD agonists as antiplatelet agents. TZDs are mainly used in the treatment of T2DM because they improve insulin sensitivity by decreasing TNF-*α* and IL-6 expression and increasing adiponectin expression [198, 199]. Troglitazone was the first PPAR*γ* agonist marketed, but was withdrawn in 2000 for causing hepatotoxicity [200, 201]. Rosiglitazone and pioglitazone are the current TZDs prescribed in T2DM and have been shown to reduce the risk of myocardial infarction and stroke [202]. As was discussed in Section 3, our laboratory demonstrated that rosiglitazone attenuates CD40L surface expression and sCD40L release from thrombin-activated platelets [63]. Downregulating the CD40/CD40L system would likely provide great clinical benefit for patients with CVD. Furthermore, 15d-PGJ_2_ was found to attenuate TXA_2_ and CD40L from thrombin-activated platelets, and prevent ATP release and ADP-induced aggregation [63]. This correlates with data from a mouse model of atherosclerosis showing that pioglitazone decreases platelet activation and delays arterial thrombus formation [138]. The PROspective pioglitAzone Clinical Trial (PROACTIVE) demonstrated that pioglitazone is protective against macrovascular events in diabetic patients [203]. Rosiglitazone was also shown to reduce serum levels of matrix metalloproteinase-9 (MMP-9), implicated in atherosclerotic plaque rupture, and the proinflammatory marker CRP in patients with T2DM [204]. Conversely, some recent studies, A Diabetes Outcome Progression Trial (ADOPT) and Diabetes Reduction Assessment ramipril and Rosiglitazone Medication (DREAM), demonstrated that rosiglitazone was associated with an increase in cardiovascular risks when compared with placebo [205, 206]. As a consequence of these recent reports that rosiglitazone may increase the incidence of myocardial infarction, a randomized, prospective, open-label trial (RECORD) was performed to assess the effects of rosiglitazone on CVD [207]. The results of this study showed a significant increase in the risk of congestive heart failure in patients taking rosiglitazone, but no significant differences in cardiovascular-related hospitalization or death. There are many limitations to the recent studies on the cardiovascular effects of TZDs, such as small sample sizes and short trials, which clearly need to be resolved before an accurate interpretation of the data can be made. In the short term, it appears that the use of rosiglitazone and pioglitazone in patients that are not at high risk for congestive heart failure is warranted [19]. However, a better understanding of the biological effects of PPARs and the cogent design of selective therapeutics without adverse effects are imperative.

One alternative may lie in a promising new class of PPAR*γ* ligands known as selective PPAR modulators (SPPARMs) that have been designed as partial PPAR*γ* agonists, retaining insulin sensitization but lacking the fat-accumulating properties of the classical TZD PPAR*γ* ligands [208, 209]. Given the success with SPPARMs in targeting insulin resistance, one can speculate that other properties of PPAR*γ* could be targeted for partial agonist design in the future to have specific antiinflammatory activity without interference of normal thrombotic benefits or risk of potential negative cardiac effects.

There are also many other PPAR candidate drugs under investigation for the treatment of metabolic syndrome. PPAR dual agonists and PPAR pan agonists are new classes of drugs that target multiple PPAR isoforms at once to produce synergistic antidiabetic and cardioprotective effects. These drugs have the potential to improve insulin sensitivity and lower triglycerides while reducing the unwanted side effects of weight gain and edema associated with the administration of fibrates and TZDs. A novel group of dual agonists have been discovered that appear to be potent agonists of both PPAR*α* and PPAR*γ*. These compounds known as alkoxybenzylglycines are synthetic tertiary amino acids, one of which has been demonstrated to have beneficial oral antidiabetic and antidyslipidemic efficacy in vivo [210, 211]. However, the therapeutic efficacy of dual and pan agonists in diabetes-associated cardiovascular risks is unknown.

PPAR*β*/*δ* agonists are being developed for their ability to treat hyperlipidemia and they have the potential to exert antithrombotic effects. It was recently published that platelets express PPAR*β*/*δ* a putative receptor for PGI_2_ whose activation inhibits platelet aggregation [64, 212–214]. Clearly, further studies are needed to address the effects that all PPAR agonists have on not only cardiovascular risks, but also on platelet activity. It appears that TZDs have potentially beneficial effects on overall cardiovascular risk. Understanding how targeting PPAR with pharmacological agents influences platelet biology will provide insight into the function of PPARs in platelets and help in designing drugs with better specificity and fewer adverse side effects.

## 6. CONCLUSION

The studies described herein illustrate a connection between PPARs and platelets that is significant in the pathophysiology of CVD. Platelets are emerging as potent immune and inflammatory mediators that both initiate early responses in the vasculature and elicit protracted responses that lead to the development of chronic inflammatory disease. Platelets contain PPAR*β*/*δ* and PPAR*γ*, nuclear receptors with known antiinflammatory functions. Thus, platelets are important contributors to CVD processes and PPARs have the ability to attenuate these processes. Platelet-derived PPARs are likely to play an important role in controlling the magnitude of a platelet-driven inflammatory response. Treatment of platelets with PPAR agonists dampens the risk of thrombus formation and attenuates increased blood levels of proinflammatory mediators such as CD40L and TXA_2_. These functions of PPARs can be exploited for the development of drugs to combat such prevalent and devastating conditions as dyslipidemia, atherosclerosis, and diabetes. Understanding the specific role of platelet-derived PPARs in the process of platelet activation attenuation is essential for intelligent prevention and management of these disease states.
